# A Cost Effectiveness Analysis of Stepped Care Treatment for Bulimia Nervosa

**DOI:** 10.1002/eat.22087

**Published:** 2013-01-28

**Authors:** Scott J Crow, W Stewart Agras, Katherine A Halmi, Christopher G Fairburn, James E Mitchell, John A Nyman

**Affiliations:** 1 Department of Psychiatry, University of Minnesota School of MedicineMinneapolis, Minnesota; 2 Stanford University School of MedicinePalo Alto, California; 3 Department of Psychiatry, Weil Cornell Medical CollegeWhite Plains, New York; 4 Department of PsychiatryOxford University, United Kingdom; 5 Neuropsychiatric Research InstituteFargo, North Dakota; 6 Division of Health Policy and Management, University of MinnesotaMinneapolis, Minnesota

**Keywords:** bulimia nervosa, cost effectiveness, cognitive behavioral therapy

## Abstract

**Background:**

The cost effectiveness of various treatment strategies for bulimia nervosa (BN) is unknown.

**Aims:**

To examine the cost effectiveness of stepped care treatment for BN.

**Method:**

Randomized trial conducted at four clinical centers with intensive measurement of direct medical costs and repeated measurement of subject quality of life and family/significant other time involvement. Two hundred ninety-three women who met DSM-IV criteria for BN received stepped care treatment or cognitive behavioral therapy. Cost effectiveness ratios were compared.

**Results:**

The cost per abstinent subject was $12,146 for stepped care, and $20,317 for cognitive behavioral therapy. Quality of life ratings improved significantly with treatment, and family/significant other time burden diminished substantially.

**Discussion:**

In this trial, stepped care for BN appeared cost effective in comparison to cognitive behavioral therapy. Treatment was associated with improved quality of life and diminished time costs of illness. © 2013 by Wiley Periodicals, Inc. (Int J Eat Disord 2013)

## Introduction

Bulimia nervosa (BN) has been the subject of active study for nearly 30 years. During that time, a number of potentially effective treatments have been developed, including various psychotherapies (provided in self-help, individual, and group formats) as well as various pharmacotherapies.^1^ As treatment research in this area has progressed, increasingly complex designs have been used that more accurately replicate real world clinical conditions. Such complex designs also raise the possibility of examining a broader range of outcomes beyond traditional efficacy measures, including parameters such as cost. Eating disorders are broadly viewed as being difficult to treat and associated with high health care costs.^2^,^3^ To date, cost effectiveness work in this field has been limited to studies using a variety of assumptions to model costs.^4^–^6^ The inclusion of cost effectiveness measures in studies of eating disorders treatment would represent a significant improvement on prior designs, since as overall health care costs grow, the pressure to examine the cost effectiveness of various interventions will increase. In addition, the costs associated with BN include both financial expenditures and time; estimates of each would be useful. The goal of this analysis was to examine the cost effectiveness of stepped care (SC) as compared to cognitive behavioral therapy (CBT) for the treatment of BN using intensive measures of direct medical costs. In addition, limited measures of time costs associated with illness were obtained. Finally, a quality of life measure was obtained at intervals throughout treatment.

## Method

### Participants

Participants in this study were 293 women with purging or nonpurging BN as defined by DSM-IV.^7^ Subjects were randomized to either cognitive behavior therapy (CBT; *n* = 147) or stepped care (SC; *n* = 146). The clinical effectiveness study is described in more detail in the accompanying paper.^8^ After a complete description of the study to the patients, written informed consent was obtained.

### Intervention

The study compared two strategic approaches to BN treatment: one began with a high intensity psychotherapy treatment (CBT), augmented as indicated with fluoxetine, reflective of what has often been referred to as current “state-of-the-art” treatment for BN. The other used a stepped series of interventions moving from less intensive and less expensive to more intensive and expensive. The CBT utilized was that described by Fairburn et al.^9^ and recently studied in two large multicenter trials for BN.^10^,^11^ Subjects receiving CBT had a total of eighteen 50 min therapy visits over 4 months of treatment.

The self-help manual in the study was also based on the CBT approach and was one developed by Fairburn.^12^ Participants randomized to self-help were seen eight times for approximately 20 min per session across the first 20 weeks. Self-help therapy focused on helping subjects learn to use the manual and providing support and encouragement.

The medication management portion of the study involved the use of fluoxetine as an antibulimic agent. Subjects could receive medication in either arm of the study via prediction of nonresponder status using a previously derived algorithm.^10^ In this algorithm, CBT participants who did not achieve at least a 70% reduction in frequency of purging by week 4 (session 6) of CBT were predicted to ultimately not achieve abstinence with CBT alone and thus were offered fluoxetine at that early stage of treatment. Fluoxetine was initiated at 20 mg per day with the maximum dose being 60 mg per day. In the SC arm of the study, participants who did not achieve at least a 70% reduction in purging frequency by week 10/session 6 were also offered fluoxetine. Twenty-minute medication management visits occurred every 2 weeks for the first five visits and then monthly. If medication was helpful but did not lead to complete abstinence in the SC arm, the option existed to continue it throughout subsequent CBT.

### Instruments

1. Eating Disorder Examination (EDE)^13^: This semistructured interview was used as a primary measure of treatment outcome, as well as to determine patient eligibility for the study. The validity and reliability of the EDE have been well documented.^14^
2. Health Care Diary: The health care diary was developed for this study to facilitate the intensive prospective assessment of health care costs reported by participants. The diary used monthly calendars for the recording of daily health care costs. At initiation and as needed throughout study participation, subjects received careful instructions on how to complete the diaries. Data were sought regarding outpatient treatments of all sorts, including emergency room visits, day and partial hospitalization usage, hospitalization, and medications. Where appropriate, information regarding duration, indication/presenting problem, and dose was sought, and diaries were reviewed for completeness by research staff at each collection point.
3. Family/Significant Other Questionnaire (FSOQ): Time costs associated with an illness (referred to as “indirect costs”) represent an important part of the burden associated with psychiatric and other illnesses. For the reasons described later, intensive measures of indirect cost were not obtained, but a limited measure was gained by using the FSOQ, developed for this study. This measured the amount of time that BN symptoms and their treatment took up for a family member or significant other nominated by the subject. The same family member or significant other completed the FSOQ for a 4-week period retrospectively at the start of the study and at the end of the first 18 weeks of treatment. Because of a concern that reluctance to reveal BN symptoms via the FSOQ might inhibit study participants from participating, completion of the FSOQ was voluntary.
4. Quality of Well Being Scale—Self administered version (QWB-SA)^15^: The QWB-SA is a well-validated measure of quality of life used in clinical cost effectiveness studies. The scale measures health-related quality of life in a broad variety of domains rather than those specifically related to BN. The QWB-SA yields a preference weighting of quality of life ranging from 0 (death) to 1 (perfect health). The QWB-SA was completed at five assessment points in the trial.

### Assessor and Therapist Training and Super vision

Self-help therapists were Master’s or Ph.D. level health care providers who did not specialize in eating disorders treatment and had received no formal training in CBT. Therapists for CBT were Ph.D. level clinical psychologists with experience and training in treating BN using CBT. Medication management and medical supervision were provided by a physician or clinical nurse specialist at each site.

A system of initial training followed by intensive supervision of psychotherapy was used. Audiotapes of therapy were used for supervision, both for on-site supervision and for study-wide treatment supervision which was conducted from one site.

Assessors with previous training and experience using the SCID-I and EDE were available at all treatment sites. Study-wide training was held at initiation of the study where assessors reviewed the EDE protocol and rated two EDE tapes, with resolution of differences in interpretation. At 3 month intervals, the data center sent a randomly selected tape from one site to the other sites to be rated by each assessor. Overall inter-rater agreement ranged from 0.91 to 0.99.

### Cost Effective Analysis

A decision was made to conduct the cost-effective analysis for this study from a third-party payor perspective, using direct medical costs for the primary analysis. Although a societal perspective analysis captures other factors (such as time costs) which are of great importance to providers, patients, and their families alike, we elected not to perform such analyses for several reasons. The first is that the time costs associated with BN are likely to be very extensive (given the time that many patients spend obtaining foods, binge eating, and purging) but measures for all aspects of this dimension have yet to be developed. Second, if such measures were available, they might likely have a very substantial therapeutic impact; in fact, a different form of self-monitoring is an integral part of CBT for BN and has been found to be a helpful aspect of treatment. While such measures would be potentially very useful, they might well confound the outcome of the trial as a whole. Third, as noted earlier, there is a widespread view that BN treatment is expensive, and as such, cost considerations play a prominent role in third party payor decisions about coverage of care, at least in the United States. Our impression is that direct costs have a substantial impact on third party payor decisions, and societal costs much less so.

For inpatient utilization, Diagnosis Related Grouping (DRG) diagnoses were derived from recording of reasons for hospital visits. DRG cost figures for 2005 were obtained from the Center for Medicare Services (CMS) website to allow for assignment of hospital costs. For outpatient and emergency room utilization, procedure codes were assigned using the 2005 Current Procedural Terminology Code Book.^16^ Then, 2005 costs from the CMS website for these services were calculated. For medication usage, lowest average wholesale price was obtained from the Red Book.^17^

The effectiveness metric choice for this analysis was abstinence at end of follow-up. Thus, the analyses yielded both mean cost of treatment for a subject in each arm, as well as the cost of achieving abstinence for one patient in each arm. This was defined as the cost-effectiveness ratio.

In cost effectiveness analysis, two sources of uncertainty need to be addressed. First, even data-based effectiveness analyses such as the one conducted here involve some assumptions, and uncertainty in those assumptions needs to be tested using sensitivity analyses. Thus, sensitivity analyses were conducted.

Second, statistical uncertainty in directly measured variables must be taken into account. This was approached using bootstrap resampling with random replacement, using 10,000 samplings with bias correction.^18^ Bootstrap estimates were used to evaluate uncertainty in the cost effectiveness ratio results because primary data were available and we did not anticipate a normal distribution for either costs or effectiveness.

Approval was obtained from the Institutional Review Board at each institution participating in this study prior to initiation of study procedures.

## Results

A total of 147 subjects were randomized to the CBT arm, and 146 to the SC arm. Demographic characteristics of the sample and overall response rates are shown in Table 1. Abstinence at the end of 1 year follow-up was observed in 18% of subjects in the CBT arm and 26% of subjects in the SC arm.

**TABLE 1:** Baseline characteristics by site and treatment group

	CBT (*N* = 147)	SC (*N* = 146)
Age	29.5(8.0)	29.8(9.8)
BMI	23.4(4.5)	23.5(5.3)
Current depression	24%	23%
Lifetime depression	62%	56%
History of AN	22%*	32%*
Personality disorder	33%	36%
% with college degree	52%	56%
Minority	16%	12%
Global EDE	3.1(1.1)	3.2(1.2)
EDE objective binges	27(25)	27(24)
EDE compensatory behaviors	44(37)	43(42)

Notes: BMI, body mass index (kg/m^2^); AN, anorexia nervosa; EDE, eating disorder examination.

Table 2 shows total costs incurred by treatment arm; costs are further broken down by category in Table 2. The costs incurred for CBT and SC differed substantially, partly as defined the study design. Utilization of medication management visits was higher in the CBT arm (*p* < .001); however, total expenditure for medications did not differ significantly between the arms (for CBT, $1,112; for SC, $904).

**TABLE 2:** Mean per subject costs by treatment condition

	CBT	Stepped Care
Total	$3,650	$3,129
CBT	1,328	509
Self-Help	0	415
Medication	1,112	904
Physician Visits	24	49
Emergency Room	465	572
Hospitalization	288	309
Individual Therapy	160	217
Group Therapy	25	41
Medication Management	248	113

Notes: Costs in 2005 U.S. dollars; CBT, cognitive behavioral therapy; SC, stepped care

**FIGURE 1:**
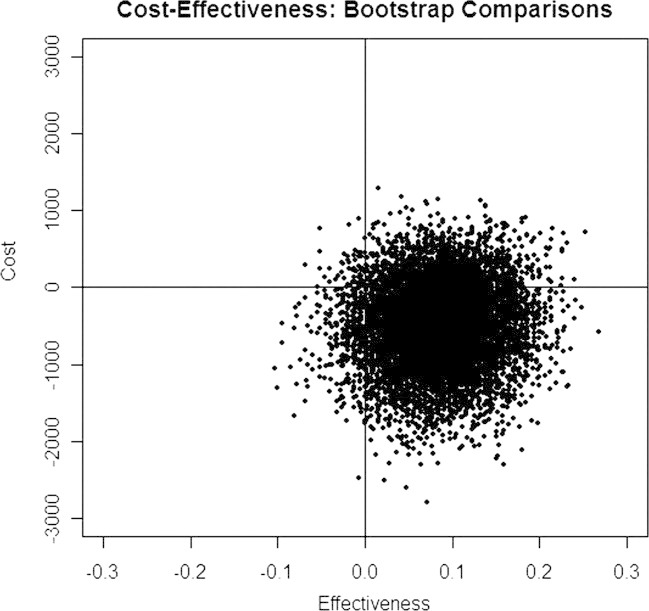
Cost effectiveness ratios for stepped care vs. CBT - bootstrapping results.

Cost effectiveness ratios representing the cost per abstinent subject at the end of observation in each treatment arm were calculated and are shown inTable 3. For CBT, the cost effectiveness ratio was $20,317 per abstinent subject. For SC the cost effectiveness ratio was $12,146 per abstinent subject.Figure 1 shows the result of 10,000 iterations of bootstrapping with random replacement comparing cost effectiveness of the two treatment arms. In 81% of bootstrapped cases, SC was both less expensive and more effective than CBT (that is, SC dominated CBT in approximately four-fifths of bootstrapped cases). Given the pattern of dominance observed, neither incremental cost effectiveness ratios nor cost-acceptability curves were calculated.

**TABLE 3:** Cost and effectiveness by treatment condition

	Cost	Effectiveness (Abstinence)	C/E Ratio
Stepped care	$3,158	0.26	$12,146
Cognitive behavioral therapy	$3,657	0.18	$20,317

A sensitivity analysis was conducted to test assumptions about unit costs. The primary analysis used CMS data for cases; re-analysis using stated unit costs (those billed to patients paying out of pocket) revealed cost effectiveness ratios of $28,132 per abstinent subject in the CBT arm, and $14,755 per abstinent subject in the SC arm.

The QWB was completed at four times during the study and the results are shown in Table 4. Overall, ratings of quality of life improved throughout the study. The difference between pre - and post-treatment ratings was statistically significant (*F* [1,853] = 43.95, *p* < .001). There was also a time by treatment response interaction with individuals achieving abstinence reporting greater improvement in quality of life (QWB-SA mean .738 [SD: 0.159]) than those who did not achieve abstinence (0.673 [SD = 0.144]; *F* [1,291] = 8.75, *p* < .003). No main effect of treatment condition was observed.

**TABLE 4:** Quality of well being scale – self administered version scores by week and treatment condition

	Mean (SD)	
Week	CBT	GSH
0	0.598 (0.101)	0.597 (0.106)
10	0.648 (0.118)	0.660 (0.126)
18	0.682 (0.137)	0.685 (0.126)
36	0.678 (0.143)	0.691 (0.139)
62	0.699 (0.152)	0.685 (0.151)

A total of 28% of subjects agreed to have a family member or significant other fill out the FSOQ and returned completed FSOQ’s from the same family member at pre- and post-treatment. As seen inFigure 2, the amount of time taken up by the family member’s illness was reported to decrease substantially over the first 18 weeks of treatment from a mean total of around 4 h to less than 1 h.

**FIGURE 2:**
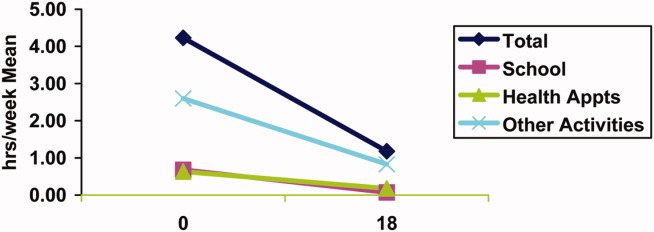
Time cost to family or significant other. [Color figure can be viewed in the online issue, which is available at wileyonlinelibrary.com.]

## Discussion

To our knowledge, this is the first BN treatment trial to use detailed prospective collection of direct health care costs in conjunction with state-of-the-art measures of clinical effectiveness. The study compared two strategic approaches to BN treatment: one began with high intensity treatment (CBT), and the other (SC) used a stepped series of interventions moving from less intensive and less expensive to more intensive and expensive. The SC intervention appeared to be more effective and cost less than the CBT approach; these findings are further supported by the results of the bootstrapping analysis, which showed that in the majority of cases, SC appeared to be both more effective and less expensive.

This study is also the first to our knowledge to report changes in quality of life measures in response to BN treatment. A number of prior studies have documented the serious impact of eating disorders on quality of life^19^–^24^ but this is the first to show that quality of life improves with treatment, and that more successful treatment is associated with a greater improvement in quality of life.

Most illnesses incur both medical treatment costs and “societal” costs such as time lost to other productive activities due to symptoms of (or treatment of) the illness. These time costs are likely quite prominent for individuals with BN and their families but until now, no attempt has been made to measure them. Findings of the limited time cost collection in this study suggest that having a family member with BN likely does incur a substantial time cost for other family members, but that time burden diminishes substantially with treatment.

There are a number of strengths and limitations to this study. The sample size, for the BN treatment study, was large. The quality of the clinical effectiveness measures was very high and the intensity of cost collection was similarly very high. Available data allowed for calculation of cost per abstinent subject; as abstinence from binge eating has considered the most desirable goal in clinical treatment (and the reporting of clinical trial results) cost per abstinent subject would be the optimal metric for such analysis. The inclusion of subjects at four centers plus the use of nation-wide standard costs (rather than regional or local costs) may increase the generalizability of the sample. Furthermore, this was in many respects an effectiveness trial, in that inclusion criteria were relatively broad and exclusion criteria relatively limited. This, too, may increase the generalizability of the findings. Furthermore, the inclusion of a year long follow-up period provides a broad picture of costs associated with BN and its treatment.

There are also several limitations to consider. First, an even longer follow-up period would have provided a still more useful picture of costs. In particular, it seems possible that treating BN, while incurring short-term costs, could substantially diminish downstream costs but this could only be examined with much longer follow-up. Second, while some measures of the time costs were collected, most of the societal costs of this illness were not examined. Third, the analysis was conducted within a clinical trial setting; how well the results would translate to real-world clinical settings is thus uncertain. Fourth, the abstinence rates seen at follow up were lower than those seen in a number of other BN CBT trials; the reasons for this are uncertain. Lower abstinence rates, however, would tend to yield higher estimates for the cost per abstinent subject.

In summary, a stepped care approach to BN treatment in this trial was more effective and cost less than CBT with the addition of fluoxetine as indicated. It is important to emphasize that this trial did not compare guided self-help with CBT; rather, the comparison was of a coherent treatment strategy involving a graded series of steps. One of the obvious public health challenges in treating eating disorders at present is the limited number of specialized programs, which results in most patients not receiving treatments with empirical support.^25^ The results of this study suggest that this SC approach may provide some potential aid for this problem by utilizing in its early stages treatments which are relatively easily disseminated. At the same time, it may provide a more time and cost effective strategy for utilization of limited resources in specialized centers.

Active drug was provided by Eli Lilly.

**Earn CE credit for this article!**

Visit: http://www.ce-credit.com for additional information. There may be a delay in the posting of the article, so continue to check back and look for the section on Eating Disorders. Additional information about the program is available at www.aedweb.org
